# Secondary traumatic stress in household members of healthcare workers in the UK: a mixed-method survey study

**DOI:** 10.1186/s40359-025-02923-6

**Published:** 2025-05-30

**Authors:** Sahra Tekin, Danielle Lamb, Talya Greene, Millie Tamworth, Dominic Murphy, Jo Billings

**Affiliations:** 1 Division of Psychiatry, University College London, 6th Floor, Maple House, 149 Tottenham Court Road, London, W1T 7NF UK; 2 Department of Applied Health Research, University College London, 1-19 Torrington Place, London, London, WC1E 7HB UK; 3 Research Department of Clinical, Educational and Health Psychology, University College London, 1-19 Torrington Place, London, WC1E 7HB UK; 4 Combat Stress, Tyrwhitt House, Leatherhead, KT22 0BX & UK; 5 The King’s Centre for Military Health Research, King’s College London, Cutcombe Road, London, SE5 9PR UK

**Keywords:** Secondary traumatic stress, Family members, Household members, Healthcare workers, Cross-sectional study, Mixed-method study, Occupational trauma, Content analysis

## Abstract

**Background:**

Due to long working hours, shifts, poor working conditions, and high risk of exposure to traumatic incidents at work, healthcare workers (HCWs) are at high risk of developing mental health and wellbeing issues. Family members and close friends of HCWs are often the primary support source for the HCWs. However, while supporting the HCWs, family members’ and friends’ mental health and wellbeing may be impacted negatively. According to the findings of previous literature, family members of other high-risk workers may experience secondary traumatic stress. To date, there has been no research focusing on secondary traumatic stress in family members and friends of HCWs.

**Methods:**

In this cross-sectional, mixed-method study, we examined secondary traumatic stress and associated factors amongst 320 household members (family members and housemates) of HCWs in the UK using the Secondary Traumatic Stress Scale. We used multivariable linear regression to examine the predictors of secondary traumatic stress, specifically sex, age, job role of the HCW, and the relationship with the HCW. Then we used content analysis of responses to open-ended questions to explore the experiences of household members in-depth.

**Results:**

33.8% of household members reported secondary traumatic stress within the severe range. Female spouses and partners of HCWs with clinical roles showed higher STS compared to male and other household members of HCWs with non-clinical roles. In our regression model, we found that being female, having a HCW household member with a clinical role, and being a spouse or a partner of a HCW were statistically significant predictors of high STS. Open-ended responses showed that household members reported that HCWs tended to be irritated, quieter/distant, anxious/stressed, in low moods, and exhausted after having a difficult day at work. These feelings and behaviours impacted the rest of the household members negatively.

**Conclusion:**

This is the first study which has examined secondary traumatic stress amongst household members of HCWs. While trying to support the HCW, household members were at high risk of developing secondary traumatic stress. There are research implications to understand HCWs’ and their household members’ experiences better, including extending current research and conducting further research exploring secondary traumatic stress in HCWs’ household members, and factors associated with it, which go beyond the demographics examined here. There are also organisational and clinical implications to protect and support both HCWs and their household members, such as improved working conditions for HCWs and carefully planned psychological support for both HCWs and their household members.

**Supplementary Information:**

The online version contains supplementary material available at 10.1186/s40359-025-02923-6.

## Introduction

Secondary traumatic stress (STS) is characterised by distress due to being exposed to the details of a traumatic event experienced by a significant other [1]. STS symptoms present as post-traumatic stress disorder symptoms including arousal, avoidance, and intrusions [2], and the current definition of PTSD has now incorporated indirect exposure to traumatic events as well [3].

Previous literature shows that family members of military personnel and veterans often report high levels of STS [1, 4, 5]. For example, in a systematic review, Diehle et al., [6] reported that spouses of help-seeking veterans with PTSD were at significantly high risk of STS. According to Diehle et al., [6], help-seeking veterans tended to talk about traumatic experiences more and that may increase the risk of STS for family members. When rates of STS have been compared between partners and parents of Dutch soldiers, researchers reported that partners showed significantly higher STS compared to parents [5]. The authors concluded that partners are usually the primary source of support as they are living in the same house with the soldiers, and thereby tended to be exposed to more details of the traumatic incidents and developed higher STS [5]. In a qualitative study conducted with wives of Israeli veterans with PTSD, some of the wives reported that while trying to help and support the veteran who suffered from PTSD, their family functionality was disrupted and the whole family started to experience the same symptoms as the veteran [7].

Whilst a growing body of research has explored the impact of military work on military personnel and veterans’ families [5, 30], relatively little research has so far been conducted with household members of other potentially high-risk occupational groups. Due to stressful, traumatic, and demanding work environments, long/unpredictable working hours and shifts, and poor working conditions [8], healthcare workers (HCWs) can be considered one such high-risk occupational group [9]. Studies conducted since the COVID-19 pandemic show that HCWs are at high risk of developing PTSD and depression [10], complex PTSD [11], moral injury and burnout [12, 13]. To cope with their work, HCWs may turn to household members for support. However, while supporting their loved ones, household members may be exposed to details of the HCWs’ traumatic experiences and are thereby at risk of being significantly impacted by their HCW household member’s job.

To date, there are only a few published research studies focusing on potential STS amongst household members of HCWs. In a qualitative study which included 14 family members and close friends of HCWs in the UK, family members and close friends of HCWs talked about being distressed by hearing about the details of traumatic incidents that the HCW experienced [14]. Similarly, in another qualitative study which was conducted in Iran, families of HCWs reported that they felt worried due to hearing the details of traumatic events experienced by the HCWs [15].

Understanding and supporting the mental health issues experienced by household members, who are often a key source of support for HCWs, is significant not only for individuals’ themselves but also for the continuity of the healthcare worker’s role and the wider healthcare system. As above, most studies related to secondary traumatic stress have mostly been conducted with veteran/military families and spouses and partners. To date, there is little qualitative literature, and no published quantitative research, reporting the degree of STS experienced by household members of HCWs.

In this study, we aimed to examine the degree of STS reported by household members (family members and housemates) of HCWs in the UK following the COVID-19 pandemic and explore associated predictors. We developed the following quantitative hypotheses: (a) spouses and partners of HCWs will report higher secondary traumatic stress scores compared to other household members (including housemates and other family members living in the same house with HCW such as children and parents), (b) household members of HCWs with clinical roles (such as doctors, nurses, and mental healthcare professionals) will report higher STS compared to household members of HCWs with non-clinical roles (such as porter, cleaner, and administrator), (c) being a spouse/partner of a HCW and being a household member of a HCW in a clinical role, will be significant predictors of higher STS. Additionally, using free text responses, we also sought to understand qualitatively how household members were impacted by their HCW household member’s work (including the impact of HCW’s traumatic work experiences on the household members) and what support household members thought would be beneficial.

## Method

This study was given ethical approval by the University College London Research Ethics committee (ID: 20221.002).

### Design and procedure

This mixed-methods study was conducted with household members (family members and housemates) of HCWs who worked during the COVID-19 pandemic in the UK. Data were collected between November 2023 and February 2024. The sample of household members of HCWs was recruited via the NHS (National Health Service) CHECK study’s email list, which includes over 23,000 HCWs in the UK. NHS CHECK is a national longitudinal study which examines the mental and physical health of staff working in 18 NHS Trusts across the UK [16].

A link to an online survey using the Qualtrics platform [17] was sent to HCWs via NHS CHECK newsletters, and we asked the HCWs to share the link with their families and housemates. The link included the participant information sheet, consent form, demographic questions, secondary traumatic stress scale, and open-ended questions. We offered a prize draw of a £50 voucher for ten participants. The questionnaires examined the demographics of the household members and the HCWs, and household members’ STS experiences. Open-ended questions helped us to explore the impact of healthcare work on household members and what support household members perceived they needed.

### Measures

#### Sociodemographic

factors were assessed with questions about household members and HCWs including sex, age, ethnicity, relationship with the household member (spouses/partner or other household members), and HCW’s job role (clinical or non-clinical).

#### Secondary traumatic stress

amongst the household members was measured using the Secondary Traumatic Stress Scale [18]. The Secondary Traumatic Stress Scale includes 17 items and aims to examine experiences of secondary traumatic stress in the last seven days, with response ranges from never (1) to very often (5) [18]. Examples of the items included in the STSS are “*I felt emotionally numb*” and “*I expected something bad to happen*” [18]. The STS scores of the participants were calculated by summing all 17 items’ scores [18]. The Secondary Traumatic Stress Scale has established good psychometrics [19, 20]. Cutoff scores for the STS were determined by following Bride et al.’s study [19] and we categorised scores as follows: little or no STS (total score < 20), mild STS (total score 28–37), moderate STS (total score 38–43), high STS (total score 44–48) and severe STS (total score > 49). The Cronbach’s alpha was 0.96 in the current study [19].

Participants were also invited to answer six optional open-ended questions; (a) Have you experienced any changes in your loved one’s behaviour when she/he has had a difficult day at work? If yes, please elaborate, (b) Have you ever been troubled by traumatic experiences of your family member/close friend healthcare worker’s work that they have shared with you? If yes, can you share with us how this has impacted you, (c) Are there any other ways in which your loved one’s work has affected you/your family or your household? If yes, can you share with us how this has impacted you, (d) Have there been any (other) positive benefits for you and/or your household/family of your loved one’s work? If yes, please elaborate, and (e) What support would you like as a family member/close friend of a healthcare worker?

### Analyses

#### Statistical analysis

Relationship with the HCW household member was dichotomised into ‘partners/spouses of HCWs’ and ‘other household members’. Healthcare workers’ job setting was also dichotomised into “clinical” or “non-clinical” based on whether the HCW had direct contact with patients to treat them or not.

To assess whether there were significant differences in the degree of STS amongst spouses/partners of HCWs vs. other household members, a two-tailed t-test was used. Similarly, the relationship between STS and age, sex, ethnicity, and HCW’s job role was explored individually using two-tailed t-tests. Then, based on the current literature (which reports them as significantly associated variables for STS in other occupational populations), age, sex, HCWs’ job roles, and relationship with the HCW were included in the multivariable linear regression model. Multicollinearity was checked. IBM SPSS version 28.0 was used to analyse the data.

#### Qualitative analyses

**Fig. 1:**
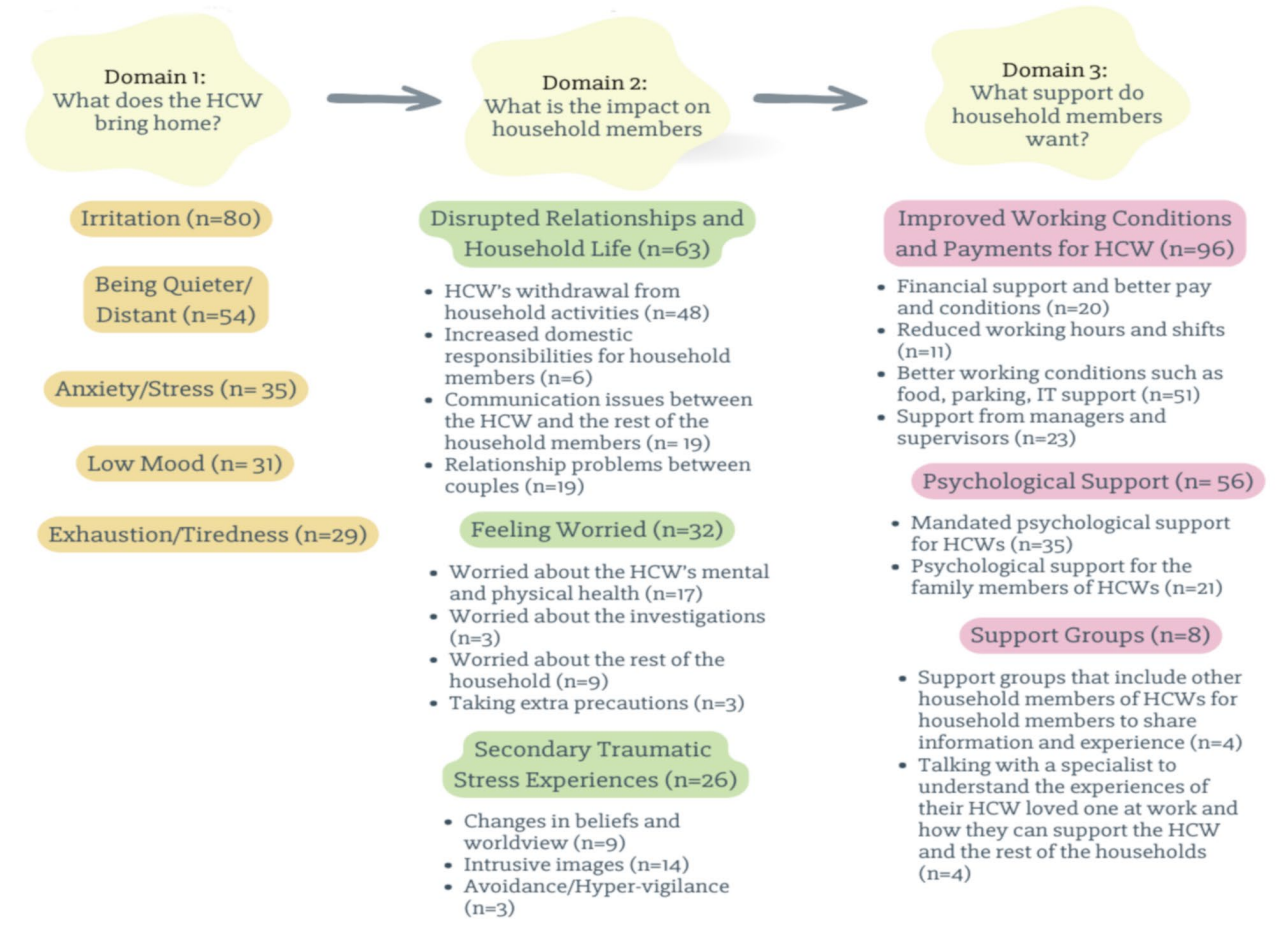
Domains, categories, subcategories, and number of participants

Content analysis was used to categorise information from the open-ended questions. Vears and Gilliam’s guidelines for qualitative content analysis were followed [21]. In this study, we employed both inductive and deductive content analysis. In the first step, to increase familiarity with the data, the first author (ST) re-read the participants’ answers multiple times, and the domains were deductively created based on the open-ended questions (See Fig. 1). In the second step, preliminary categories based on the main content of the participants’ comments were created inductively. In the third step, text under each preliminary category was analysed line by line and preliminary subcategories were identified. The initial categories and subcategories were then discussed with JB as a fourth step. A proportion of the data was independently categorised by a second coder (MT) using the identified categories and sub-categories. There was no disagreement between the coders. Finally, the categories and subcategories were reported narratively.

## Results

Six hundred and sixty-six participants responded to the study invitation, of whom 383 completed the questionnaires. Fifty-one participants who lived in different households from the HCW and twelve participants who did not complete the STSS were excluded. In total, 320 participants completed all relevant questions and were included in the statistical analyses.

Out of 320 participants, 142 household members were female, and 171 household members were male. Seven household members preferred not to share their sex. Two hundred and seventy-four of the participants described their ethnicity as white. Out of 320 participants, 258 were spouses or partners of HCWs. Two hundred and thirty-found of the participants reported that their HCW loved one worked in a clinical role with direct contact with patients. See Table 1 below for the participants’ characteristics.

**Table 1:** Demographic characteristics of survey participants

	*n* (%)
**Sex of the Household Members**	
Female	142 (46%)
Male Prefer not to say	171 (52%) 7 (2%)
**Age Range**	
18–24	29 (9%)
25–34	59 (19%)
35–44	80 (25%)
45–54	75 (23%)
55+	77 (24%)
**Ethnicity of the Family Member**	
White	274 (85.5%)
Other	46 (14.5%)
**Relationship with the HCW**	
Spouses/Partners - *Husband* - *Wife* - *Partner (gender not stated)*	258 (79%) *120* *63* *75*
Other Household Members - *Children* - *Sibling* - *Friend (Housemate)* - *Parent*	62 (21%) *39* *4* *11* *8*
**HCW’s Role**	
Clinical Role - *Doctor* - *Nurse* - *Mental Healthcare Worker* - *Paramedic* - *Physiotherapist* - *-Occupational Therapist*	234 (74%) *50* *105* *48* *3* *11* *17*
Non-clinical Role - *Administrator* - *Catering* - *Chaplain* - *Cleaner* - *Healthcare Assistant* - *Porter* - *Engineer* - *Manager*	86 (26%) *43* *2* *3* *4* *1* *1* *3* *29*

### Quantitative analysis

A third of the participants (*n* = 108) reported severe secondary traumatic stress scores (*M* = 61, *SD* = 8.2). Around 10% of the participants reported high (*n* = 33) secondary traumatic stress scores, 16% reported moderate (*n* = 51), 23.8% reported mild (*n* = 76), and 23.1% reported low or no STS (*n* = 74) scores.

To test our first hypothesis, we conducted a two-tailed t-test, and we found that female household members (*n* = 142) reported significantly higher secondary traumatic stress (*M* = 44.1, *SD* = 17.8) compared with male (*n* = 171) household members (*M* = 40.3, *SD* = 15.1), *t*(311) = 2.03, *p* <.005. We also found that spouses and partners of HCWs reported significantly higher STS (*M* = 44, *SD* = 16.6), compared with other household members who reported lower STS (*M* = 35, *SD* = 13.5), *t*(318) = 3.85, *p* <.001. Household members of HCWs with clinical roles showed significantly higher STS (*M* = 44.1, *SD* = 16.7) compared to household members of HCWs with non-clinical roles (*M* = 36.1, *SD* = 14.1), *t*(318) = 3.7, *p* <.001. Household members’ secondary traumatic stress scores did not significantly differ based on their ethnicities or their ages.

Bivariate correlations were run independently amongst the six variables (sex, age, ethnicity, HCW’s job role, relationship with the HCW, and STS) (See Table 2). There was a positive correlation between STS scores and being female, being a spouse or partner of an HCW, and living with a HCW with a clinical role. There was no significant correlation between STS and ethnicity.

Multivariable linear regression analysis was conducted to examine the predictors of STS, specifically sex, age, job role of the HCW, and the relationship with the HCW. The overall model was statistically significant (*F*(5, 314) = 9.53, *p* <.001), and accounted for approximately 36% of the variance in STS scores. As shown in the Table 3, sex, HCW’s job role, and the relationship with the HCW were significant predictors of STS. Specifically, STS was positively associated with having a HCW household member with a clinical role (*r*(314) = 0.215, *p* <.001) and being a spouse/partner of a HCW (*r*(314) = 0.246, *p* <.001). Multicollinearity was assessed using Variance Inflation Factors (VIFs). All VIF values ranged between 1.02 and 1.8, well below the commonly accepted threshold of 5, indicating that multicollinearity was not a concern in this model. In summary, based on the multivariable linear regression models’ findings, being a spouse/partner of a HCW and living with a HCW with a clinical role were predictors for high STS, after controlling for sex and age (See Table 3).

**Table 2:** Variables and correlations with each other

Variables	STS	Sex	Age	Ethnicity	HCW’s Job Role	Relationship with the HCW
**STS**						
**Sex (Female)**	0.11*					
**Age**	0.05	0.16**				
**Ethnicity (White)**	0.08	0.09	0.03			
**HCW’s Job Role (Clinical )**	0.20**	0.01	0.02	0.02		
**Relationship with the HCW (Spouses/Partners**	0.21**	0.39**	0.18**	0.14**	0.01	

*p <.05, **p <.01

**Table 3:** Multivariable linear regression model (*n* = 320)

Predictor variable	B	SE	β	t	*p* Value
**Sex (Female)**	7.2	1.9	0.22	3.8	< 0.005*
**Age (in years)**	2.4	2.2	0.062	1.9	0.44
**HCW’s Job Role (Clinical)**	7.8	2.01	0.205	3.9	< 0.001**
**Relationship with HCW (Spouse/Partner)**	11.1	2.5	0.265	4.5	< 0.001**

LL: Lower Limit, UP: Upper Limit; *p <.05, **p <.01, SNS: Statistically not significant

#### Model summary

F(5, 314) = 9.53, *p* <.001, R² = 0.36, Adjusted R² = 0.12.

### Qualitative content analysis

Out of 332 participants, 267 household members provided information in response to the open-ended questions. We deductively organised our analysis into three domains, within which we identified a number of inductive categories and subcategories (See Fig. 1).

#### What HCWs bring home

Out of 267 participants who responded to this open-ended question, 102 household members reported that when the HCW had had a difficult day at work, that they experienced changes in the HCW’s behaviour. For example, 80 household members observed that the HCW was more irritated, angry, and easily annoyed, after a difficult day at work compared to other days. Additionally, 35 household members reported that the HCW was more anxious, 31 of them reported that the HCW had lower mood and 29 household members observed that the HCW was more exhausted. Fifty-four household members of HCWs pointed out that after a difficult day at work, their HCW loved one tended to be quieter and distant from the rest of the household members. Some noted that on those difficult days, HCWs tend to be less empathetic and compassionate to the rest of the household. For example,

*“[She] is struggling to sleep/worrying about specific patients and/or her clinical decision/action/inaction. If she feels like her work is incomplete or her clients are not ok until she next sees them she is worried and so grumpy and cranky with me.” (A partner of a nurse)*.

#### Impacts of healthcare workers’ distress on household members

Out of 267 household members who answered this question, 130 household members reported that the HCW’s work had affected themselves and the rest of the household in the ways detailed below.

#### Disrupted relationships and household life

Sixty-one household members said that their household life and relationships were disrupted due to the HCW’s job. For example, 48 household members pointed out that as a consequence of their HCW household member coming home from work and being upset, frustrated, or low in mood, that he/she did not want to participate in household activities. A male partner of a nurse shared his experiences below:

[She is] in low mood, short tempered, doesn’t want to be around others, only watches TV/reads a book alone. She does not want to participate in any family activities after work.

Similarly, 24 household members reported that the demanding nature of the HCW household members’ work impacted on the rest of the household, making it difficult to plan household activities. For instance, a female partner of a healthcare assistant reported that:

Work schedules change every 3 months to suit needs of the hospital which makes planning my own work schedule and our joint social events much more complicated. The lack of staff at my partner’s workplace also means there are only two points in the year they can take off a whole week of work at one time which limits how we plan family vacations and time off.

Similarly, the male husband of an administrator shared his experiences with these words;

I do feel like my husband had a choice to go into the work he has, but I don’t have a choice around how it impacts us as a family or as a partnership. He chose that work, I didn’t, and so when he is then less available at home emotionally or socially that leaves a greater burden on me and also means we are losing out on the best of him because work gets that a lot of the time.

Due to workloads and long shifts, HCWs tended to come home mentally and physically tired. Six household members specifically reported that as a result of this they had to take on more domestic responsibilities. For example, the female wife of a psychologist shared her experiences below:

*“[Psychologist HCW] required to work overtime and thus impacting on childcare*,* household tasks. Their work takes priority*,* so family life and school pick up arrangements are changed in response to that”.*

Six household members reported that HCWs struggled to separate home and work life. Due to feeling like their HCW household member was distant of cut off, four participants described how this made them feel like they were not a priority for the HCW. Nineteen household members reported that the HCWs’ feelings and behaviours created communication problems with other household members. For example,

*“I feel like my partner is overworking and prioritising work over me” (Female partner of other allied health professional)*.

*“They did not share their experiences*,* they felt they needed to be strong and never got help from us or therapy. It ruined our family. We never speak at home*,* we never hug*,* our lives as a family are secondary to their work” (Son of a consultant doctor)*.

Specifically, 19 spouses and partners reported relationship problems due to a lack of intimacy, empathy, and compassion. For example, the wife of a doctor reported that.

*“My husband is frequently angry and feels miserable. Our daughter has had depression as a result. My husband all but stopped having sex*,* only watches TV at home and before going to work” (Female wife of a doctor)*.

#### Feeling worried

Thirty-two household members reported that they had felt worried due to the nature of the HCW’s job. For instance, 17 household members pointed out that they were worried about their HCW household member’s mental health and physical health.

*I have been worried for her physical and mental wellbeing. Some examples: Twice a week for a year she worked alone late at night running a clinic with patients who were often angry*,* with no panic button and no way to escape the room*,* and no action from her superiors when the issue was raised. She has frequently been verbally*,* sexually*,* and physically attacked by patients on the ward*,* sometimes with weapons*,* and it being laughed off by other staff. During covid she was sent onto ICU without a mask which fitted*,* which resulted in her getting covid. Repeatedly being discriminated against by her superiors due to her race and/or gender. I am convinced that one day this job will kill her*,* if not directly then by driving her to suicide. (Male husband of a pharmacist)*

Three of the spouses and partners of HCWs reported that HCWs were working too long and that this could make them open to making mistakes. For this reason, some of the household members pointed out that they were worried about potential litigation. For example, a female wife of an allied health professional shared her feelings with us with these words:

Patient complaint police involved poorly handled by trust husband was distraught and felt no one believed him, and effect on him and kids. [I felt] fear of jail and there was a lack of belief and support from trust.

Nine household members stated that they were also worried about the mental and physical health of the rest of the household members. For example, a female partner of an allied health professional said that due to the infection risk that the HCW could bring home, she was worried and taking more precautions in terms of hand hygiene and general increase in anti-bacterial cleaning. Additionally, some household members reported that they were worried about their own safety due to their HCW household member’s job. For example,

*“I concern about mentally unstable colleague who knew where we lived. Made me worry for our safety.” (Female partner of a psychologist)*.

#### Secondary traumatic stress experiences

Out of 266 household members who responded to this question, 102 household members reported that they had been troubled by traumatic experiences related to their HCW household members’ work that the HCWs had shared with them. For example, 26 household members described experiences consistent with secondary traumatic stress. Several stated that there was a real physical threat for HCWs, and this impacted the whole household. Nine household members reported that after hearing about the traumatic experiences of HCWs, they started to question humanity and the safety of the world, and reported changes in their beliefs and world view. For example,

*“They have had to work with some very difficult cases which make me worried about humanity.” (A male husband of a psychologist)*.

*“[I feel] distress when I remember the stories of her patients and their lives and/or recount the traumatic stories of her clients (NB her client group is the homeless population). Worry about the state of the NHS and its ability to provide for me and my family. Overwhelmed by state of the world that’s resulting in her client’s situations.” (Female partner of a nurse)*.

HCWs’ traumatic experiences triggered household members’ previous traumatic experiences as well. For example, a nurse’s male partner who also worked in PICU stated that his partner’s experiences were triggering for him.

She would often offload her stress when she comes home, which is fine with me, although I had worked on a PICU before, so could touch lightly on my own trauma from the ward.

The female friend of a chaplain also reported that,

Talking about people’s death through cancer, remind me of my grandfather’s death when I was a child aged 4.

Fourteen household members reported that they experienced intrusive, repetitive images in their minds and associated distress related to the traumatic experiences their HCW household member had told them about.

*“Some of the images and stories [that the doctor partner shared] are quite shocking and play over in my mind.” (Female partner of a doctor)*.

*“Significantly concerned about partner’s safety and mental wellbeing. Incidents that I’ve been told about coming back to me at random moments and triggering emotional response - fear*,* sadness*,* hopelessness*,* anxiety.” (Female wife of an occupational therapist)*.

Three household members reported avoidance and hypervigilance related to their HCW loved one’s traumatic experiences. For instance, a male husband of a nurse shared his experiences after the HCW’s traumatic experiences with these words;

Someone made threats to kill him, this was a problem for many months. He works also close to where we live. So, we all became hypervigilant, would not go out for meal or socialising, because we think that that person can kill us too.

#### Support that household members need

This question related to what kinds of support participants thought could be helpful for household members and was answered by 130 household members, as detailed below.

#### Improved working conditions and pay

Ninety-six household members reported that the priority in supporting the mental health and wellbeing of the household members (including the risk of developing mental health issues) was primarily for HCWs to be better looked after, and that their working conditions and pay needed to be improved. Some of the household members reported that when HCWs’ basic needs are met, the risk of developing mental health and wellbeing issues for the household members may also be reduced. Twenty household members pointed out that providing financial support and better remuneration was required to support healthcare workers and their households. Fifty-one household members reported that HCWs’ working environments needed to be improved. Some of the household members reported that the unsafe work environment of the HCWs impacts their mental health and wellbeing in a negative way as well. For example, a male husband of a nurse shared his thoughts with these words;

Generally speaking I worry about her safety at work and being attacked/abused by patients/clients. The facilities are not there to provide a safe environment and the nature of her job means her wellbeing and physical safety are suffering detriment. There is also not adequate facilities to work remotely without causing back pain and she doesn’t feel able to ask. I would like better equipment, IT, money and proper cover so she’s not feeling guilty when she takes annual leave and her clinics aren’t covered by anyone. I also feel very angry with the lack of respect and pay that nurses get compared to doctors, it’s hierarchical where nurses are bearing huge amounts of emotional/mental burden and doing more and more that doctors do but they are STILL seeming as ‘less’ than a doctor.

Additionally, household members also pointed out that working conditions and lack of resources need to be addressed. A male husband of a psychotherapist said that,

A part time job should be part time and not eat into all hours! NHS staff are HUGELY underpaid, undervalued and way way way OVERWORKED.

Twenty-three household members stated that their HCW loved one was not getting enough support from their supervisors and managers and this needed to be improved. For instance,

*“The best support I can have [as a partner of a HCW] is to make sure they are well supported and managed.” (Male partner of other allied health professional)*.

#### Psychological support

Fifty-six household members commented that their HCW loved ones and the household members needed psychological support. Thirty-five household members suggested that mandatory psychological support would be helpful for HCWs, and indirectly household members.

*“More support for my partner in work with coping with workplace incidents*,* inconveniences and general stress. An outlet in work for raising issues that have caused grief*,* how to overcome them and avoid a repeat. Not leaving the issue to linger or grow out of control.” (Male partner of a nurse)*.

Twenty-one participants also suggested that household members would benefit from psychological support as they are also impacted psychologically by supporting their HCW loved ones. A female partner of a physiotherapist wrote,

My partner is a physiotherapist and worked on the ‘frontline’ during COVID-19. He came home most days saying he had a good day. It was only months later that he disclosed he had PTSD and felt he needed counselling. I felt that I had failed him, and the stories he shared with me of patients dying were deeply distressing. Now, I need psychological support too.

#### Group-based support

Four household members suggested that they would benefit from group-based support including other household members of HCWs. They felt this would facilitate information sharing and social support for each other. For example, the wife of a psychologist stated that,

It could be nice to have a social group including other family members [of other HCWs] or a support group for those impacted.

Four household members pointed out that they sometimes felt like they did not understand what their HCW household members experienced and they did not best know how to support the HCW loved one, the rest of the household, and themselves. For this reason, these participants suggested that receiving professional support could be helpful.

*“The NHS do not give enough recognition to the families behind the worker. For example*,* when the workers have a tough time at work*,* and they cannot divulge all the confidential information. It’s like trying to support them without having someone who could give me some advice on how to deal with things that might make things better for him or for the rest of the family.” (Male husband of an administrator)*.

## Discussion

In this mixed-method survey study we aimed to investigate the degree of STS experienced by household members of HCWs in the UK after the COVID-19 pandemic and identify predictors of STS. Additionally, we sought to qualitatively explore the impact of HCW work on household members and what support household members thought would be helpful. We found that 33.8% of the household members experienced STS within the severe range. Female spouses/partners of HCWs with clinical roles reported higher STS compared to males other household members and HCWs with non-clinical roles. Based on the findings from our multivariable linear regression model, being a spouse/partner of a HCW and having a HCW with a clinical role were significant predictors for high STS, after controlling for sex and age. We also found that according to household members, HCWs tended to be irritated, quieter/distant, anxious/stressed, in low moods, and exhausted after having a difficult day at work. These feelings and behaviours impacted the rest of the household members negatively. Household members reported disrupted relationships and household lives, worrying about HCWs and the rest of the household members’ mental and physical health. Our qualitative findings also help to elaborate on the quantitative findings. For example, household members reported secondary traumatic stress experiences related to hearing about their household members’ work, and pointed out that the HCWs and their household members need support.

The findings of our study demonstrate that STS amongst household members of HCWs may be similar to family members of other high-risk occupational groups. For example, in a systematic review which included 16 qualitative and quantitative studies that explored the impact of occupational trauma on first responders and their families, Casas and Benuto [22] reported that spouses and partners of first responders may experience STS and also experienced their partners’ problematic behavioural and emotional responses at home. Whilst spouses and partners would provide support for their first responder spouse, sometimes this came at a cost to their own mental health and wellbeing. Also, similar to our findings, Casas and Benuto [22] reported that while supporting their first responder spouses, they tended to be exposed to the details of traumatic job experiences which increased the risk of developing mental health and wellbeing issues.

To the best of the authors’ knowledge, the was the first study that has explored partners and spouses of HCWs compared to other household members. In our study we identified significantly higher STS in spouses and partners of HCWs compared to other household members of HCWs. This is consistent with research with other high risk occupational group workers’ families. In a survey conducted with 708 partners and 332 parents of Dutch military personnel, partners reported higher STS scores compared to parents [5]. The researchers of this study concluded that partners tended to be a primary source of support and therefore were exposed to more detail of the soldiers’ traumatic experiences, and hence partners tended to develop higher STS [5]. Similarly, in a longitudinal survey study with police officers and their spouses, spouses and partners of police officers with higher PTSD rates showed high STS scores [23].

The secondary trauma experiences within the household members of HCWs that we have reported with our quantitative data were elaborated on by survey participants’ responses to open-ended questions. Some household members illustrated how their beliefs and worldview were changed in a negative way due to hearing about HCWs’ work experiences. In their cross-sectional survey study with partners of emergency responders, Alrutz et al. [24] reported that 20% of partners experienced intrusive thoughts, arousal, and avoidance due to hearing about the traumatic experiences of the responder. In our study, fourteen household members reported intrusive images, and three spouses/partners specifically described avoidance/hypervigilance related to the HCW’s traumatic work experience. This again may be explained by workers tending to share their traumatic experiences in detail with their spouses and partners more than other family members [5].

We also found that household members of HCWs with a clinical role reported higher STS compared to household members of HCWs with a non-clinical role. This finding is consistent with the current literature which shows that family members of high-risk workers who are involved in life-threatening duties are at high risk of developing mental health and wellbeing issues. For example, in a survey study conducted with 144 HCWs and their 135 children during the COVID-19 pandemic in Turkey, researchers reported that children of HCWs who had direct contact with patients during the pandemic showed higher anxiety compared to children whose parents were not working in a directly clinical role [25]. Similarly, according to the findings of a cross-sectional study, which was conducted with parents of Israeli war veterans to understand parents’ STS experiences, parents of veterans who were directly involved in the war showed significantly higher STS compared to parents of veterans who did not directly participate in the war [30].In the current study, household members’ observations about HCWs’ mental health and wellbeing being negatively impacted by their work were also consistent with other studies [10, 12]. Research conducted during the COVID-19 pandemic reported that HCWs reported feeling overwhelmed [12], with high levels of anxiety, depression and post-traumatic stress symptoms [10]. In our study, household members’ observations about HCW’s mental health and wellbeing were similar to the other qualitative findings in the literature. Similarly, we found that household members observed that after experiencing a difficult day at work, their HCW household member tended to be irritated, quieter, and distant at home. Additionally, household members reported that their HCW household members tended to be lower in mood, more anxious, and exhausted after a difficult workday. Further, some household members of HCWs reported that their family life was often disrupted due to the HCWs’ withdrawal from family activities and HCWs’ long working hours. These findings are consistent with a small body of relatively recently published research which has also explored the impact of high-risk occupational roles on families. In a systematic review of qualitative research which focused on the experiences of family members of military personnel with PTSD, researchers reported that family life was often impacted due to military personnel’s work [26]. For example, family members reported that their military family member was often unable to participate in family life, and frequently missed family activities due to deployments [26]. Similarly, in a cross-sectional study conducted with 515 partners of police officers in Portugal, Costa and Silva [27], reported that their family activities and joint social life were impacted negatively due to police work. In a qualitative study with fourteen family members and close friends of HCWs in the UK during the COVID pandemic, Tekin and colleagues [14] reported that family members tended to take on more domestic responsibilities at home to support HCW and reported an increased workload at home for themselves.

In this study, we identified that household members were worried about HCW’s mental and physical health as well as other household member’s health, regardless of the COVID-19 pandemic. Likewise, in a qualitative study conducted with family members and close friends of HCWs in the UK during the COVID-19 pandemic, families and friends expressed their worry about the HCW’s health in addition to the rest of the household’s health (Tekin et al., 2022). In the current study, we found that household members were also worried about the risk of investigations and litigation for HCWs.

We also asked participants what kinds of support they thought household members needed. The majority of participants pointed out that taking care of the HCW, such as providing a better work environment, pay, and managerial support, were key elements in supporting the whole household. This was consisted with a meta-synthesis study related to experiences and needs of HCWs during the pandemics; including the COVID-19 pandemic, that concluded that HCWs prioritise systematic changes and improvements in their work environments (such as manageable workloads and support from supervisors, managers, and peers) [28]. Psychological support for both the HCW and household members was also suggested as a potential means for improving the mental health and wellbeing of whole household. Some household members also suggested that providing support groups that included household members of other HCWs to share information and experiences may be helpful for some household members, particularly as some participants pointed out that they struggled to understand their loved one HCW’s job-related experiences.

### Strengths and limitations

There are a number of strengths of this study. Firstly, to the best of our knowledge, this is the first study to report on the degree of STS, associated predictors, and experiences of household members of HCWs, following the COVID-19 pandemic. Secondly, the research team behind this study included senior clinical academics, mid and early-career researchers, bringing a diversity of experiences and perspectives to this study. Additional coders were included in the qualitative content analysis to increase the trustworthiness and the quality of the qualitative analysis. Thirdly, we achieved a reasonably large sample compared with other studies on household members of other high-risk occupational groups.

There are also a number of limitations to this study. Firstly, due to the cross-sectional nature of the survey design, our findings reflect a single point in time and cannot provide information about the STS experiences of HCWs’ household members over time. The sample is also a convenience sample, creating some limits to generalisability and external validity [29]. Secondly, our study advertisement was shared in the NHS CHECK newsletter to recruit participants. NHS CHECK participants (HCWs’ themselves) were invited to share our study’s details with their household members. Therefore, we do not know how many NHS CHECK participants had relevant household members and how many chose to share the information about the research. For this reason, it is impossible to calculate the response rate of this study and difficult to determine how representative the responses are of the whole target population. Thirdly, STS was measured using the STSS, which is based on DSM-IV criteria for PTSD and only asks questions about experiences within the last seven days [18]. Fourthly, data were self-reported, and we have no means of corroborating reported rates of STS. Finally, there was an imbalance between some groups within the sample in terms of size. For example, while 263 participants described themselves as spouse or partner of a HCW, only 69 of the participants were other household members of HCWs. Additionally, the qualitative study reports themes and sub-themes based on a sub-sample of the overall survey respondents. For example, only three household members reported avoidance and hypervigilance experiences. For this reason, this may limit the generalizability of those experiences.

### Implications

Based on household members’ needs highlighted in the open-ended questions and synthesis of the quantitative and qualitative findings from this study, we have highlighted organisational, clinical, and research opportunities to potentially improve the mental health and wellbeing of household members of HCWs. In terms of organisational support, according to findings of a systematic review and meta-synthesis of 46 studies which focused on the experiences and views of the HCWs in different pandemics including COVID-19, SARS, and Ebola, Billings et al., [28] recognised that increased working hours and shift patterns could have a detrimental impact on HCW’s wellbeing. The findings of the current study extend this by demonstrating that such working patterns can have a detrimental impact on HCW’s families as well as on the workers themselves. Prioritising pay and working conditions of HCWs, not only to maintain the healthcare force, but also to potentially protect their families and support systems who are indirectly impacted by healthcare work, and further maintain support for the HCWs themselves.

In terms of clinical implications, providing access to evidence-based psychological support, including timely assessment and treatment, may be beneficial to HCWs, directly, and indirectly, of benefit to their families. However, potential issues with confidentiality and risk management need to be considered. Occupational Health Services in healthcare settings should make sure that they are asking questions about the impact on family members if a HCW has been affected by a traumatic incident, as well as exploring the impact on the HCW themselves. Psychological support could be extended to household members to identify their needs and to provide or signpost to evidence-based treatment where needed. We also found that some household members were struggling to understand their HCW loved one’s experiences at work, and therefore services could consider piloting informational resources or peer support groups which include household members of other HCWs to share information and experiences and to evaluate how helpful this might be. Additionally, induction programmes for families and household members of HCWs who have newly joined NHS Trusts may be helpful for families to understand their HCW loved one’s experiences.

In terms of research implications, secondary traumatic stress amongst household members of HCWs is a new topic in clinical research. For this reason, more research needs to be conducted related to secondary traumatic stress and associated factors, in addition to the demographics of household members of HCWs explored here. The data for this study was collected via NHS CHECK which was developed in the COVID-19 pandemic. Even though the questions were not specific to COVID-19 experiences, it has likely had an influence on participants’ answers. More research is required to explore household members’ experiences after the pandemic and if family members’ mental health and wellbeing issues continue to persist in the post-pandemic context. (See Appendix 1 for the potential implications and recommendations)

## Conclusion

To date and to the best of the authors’ knowledge, this is the first study which has examined STS amongst household members of HCWs. Similar to other studies which have focused on family members of other high risk occupational groups, we found that in trying to support their HCW loved ones, household members of HCWs were at high risk of developing STS. In addition to the findings of the previous studies, we also found evidence that STS was prevalent within the family members of HCW and that STS appeared to be linked not only to hearing about the experiences of the HCW, but also more practical issues related to occupational stresses (such as work patterns, work load pay and conditions). There are organisational, clinical, and research implications to protect and support both HCWs and their household members, including improving the working conditions of the HCWs, providing psychological support to HCWs and their household members, and conducting more research to understand household members’ experiences and needs.

## Electronic supplementary material

Below is the link to the electronic supplementary material.

Supplementary Material 1

## Data Availability

The datasets generated and analysed during the current study are not publicly available due to the personal and sensitive content of the participants’ accounts (such as email addresses) but are available from the corresponding author (ST) on reasonable request.

## Abbreviations

HCWs: Healthcare Workers
NHS: National Health Service
PTSD: Post-traumatic stress disorder
STS: Secondary Traumatic Stress
STSS: Secondary Traumatic Stress Scale
