# Supplementary material for: Secondary traumatic stress in household members of healthcare workers in the UK: a mixed-method survey study
Source: BMC Psychol. 2025 May 30;13:584. doi: 10.1186/s40359-025-02923-6 (PMC12125900; doi:10.1186/s40359-025-02923-6)
Supplement: Supplementary file 1 — Supplementary Material 1 [file 40359_2025_2923_MOESM1_ESM.docx]

**Appendices**

**Appendix 1:**

|  | |
| --- | --- |
| **Implications** | **Recommendations** |
| **Organisational Implications** | |
| 1. Providing better working conditions | Providing better working conditions for HCWs (such as access to healthy food, IT support), better pay and conditions may be critical for both HCWs’ wellbeing but also that of their household members. |
| **Clinical Implications** | |
| 1. Psychological Support  - For HCW - For household members | Carefully planned psychological support should be delivered to both HCWs and could be extended to their household members. |
| 1. Information and support | Providing information for families and household members and potentially piloting sources of peer-based support. |
| **Research Implications** | |
| 1. More research about mental health and wellbeing of household members of HCWs | Extending current research and conducting further research exploring secondary traumatic stress in HCWs’ household members, and factors associated with it, which go beyond the demographics examined here. |
| 1. Conducting further research in the post COVID-19 pandemic context | Further research in the post COVID-19 pandemic context is required to explore whether similar findings are replicated and whether new challenges are identified. |
